# Shared Cognitive Impairments and Aetiology in ADHD Symptoms and Reading Difficulties

**DOI:** 10.1371/journal.pone.0098590

**Published:** 2014-06-02

**Authors:** Celeste H. M. Cheung, Alexis C. Fazier-Wood, Philip Asherson, Fruhling Rijsdijk, Jonna Kuntsi

**Affiliations:** 1 MRC Social, Genetic and Developmental Psychiatry Centre, Institute of Psychiatry, King's College London, London, United Kingdom; 2 Human Genetics Center, Division of Epidemiology, Human Genetics and Environmental Science, School of Public Health, University of Texas Health Sciences Center at Houston, Houston, Texas, United States of America; Center for BrainHealth, University of Texas at Dallas, United States of America

## Abstract

**Background:**

Twin studies indicate that the frequent co-occurrence of attention deficit hyperactivity disorder (ADHD) symptoms and reading difficulties (RD) is largely due to shared genetic influences. Both disorders are associated with multiple cognitive impairments, but it remains unclear which cognitive impairments share the aetiological pathway, underlying the co-occurrence of the symptoms. We address this question using a sample of twins aged 7–10 and a range of cognitive measures previously associated with ADHD symptoms or RD.

**Methods:**

We performed multivariate structural equation modelling analyses on parent and teacher ratings on the ADHD symptom domains of inattention and hyperactivity, parent ratings on RD, and cognitive data on response inhibition (commission errors, CE), reaction time variability (RTV), verbal short-term memory (STM), working memory (WM) and choice impulsivity, from a population sample of 1312 twins aged 7–10 years.

**Results:**

Three cognitive processes showed significant phenotypic and genetic associations with both inattention symptoms and RD: RTV, verbal WM and STM. While STM captured only 11% of the shared genetic risk between inattention and RD, the estimates increased somewhat for WM (21%) and RTV (28%); yet most of the genetic sharing between inattention and RD remained unaccounted for in each case.

**Conclusion:**

While response inhibition and choice impulsivity did not emerge as important cognitive processes underlying the co-occurrence between ADHD symptoms and RD, RTV and verbal memory processes separately showed significant phenotypic and genetic associations with both inattention symptoms and RD. Future studies employing longitudinal designs will be required to investigate the developmental pathways and direction of causality further.

## Introduction

Attention deficit hyperactivity disorder (ADHD) and reading disability are strongly heritable, complex neurodevelopmental disorders that frequently co-occur [1]–[4]. Sibling and twin studies indicate that the phenotypic association between ADHD and reading difficulties (RD) is largely attributed to shared familial/genetic influences [1], [3], [5], [6]. Of the two ADHD symptom domains of hyperactivity-impulsivity and inattentiveness, RD shows stronger phenotypic and genetic associations specifically with inattention symptoms, compared to hyperactivity-impulsivity symptoms [3], [5]–[7].

Linking the familial risk factors in ADHD to cognitive impairments, we obtained evidence in sibling-pair analyses for two familial cognitive impairment factors in ADHD [8]. The first and larger familial factor captured familial influences on RT variability (RTV), and is separated from the second familial factor which captured familial influences on commission errors (CE) and omission errors (OE) on a Go/No-Go task. Applying the same analysis approach to an independent dataset of ADHD and control sibling pairs, with different cognitive and motor tasks, again two familial factors emerged where familial factor loading on ‘intra-individual variability’ was separate from those on working memory (WM) [9]. Overall, the findings from the sibling studies on children and adolescents with ADHD indicate two familial cognitive impairment factors in ADHD, the first capturing slow and high variable responses and the second capturing aspects of executive functioning, both of which largely separate from familial influences shared between ADHD and IQ.

By mapping the aetiological factors underlying ADHD-related cognitive impairments onto those of the two ADHD symptom domains separately using a population sample of twins, we further demonstrated that RTV and CE reflect different genetic relationships to the two ADHD symptom domains [10]. While RTV showed substantial genetic overlap particularly with inattentiveness, CE showed little genetic overlap with either hyperactivity-impulsivity or inattentiveness.

Similar to ADHD, individuals with RD also show impairments in multiple domains of cognitive functions including verbal WM [11]–[16], RTV [11], [17]–[19] and processing speed [16], [20], [21]. However, RTV and verbal working memory have been more extensively studied in ADHD. Findings on response inhibition have, however, been inconsistent [15], [16], [22]. A sibling study indicated significant shared familial influences on RD with executive functioning and motor vulnerabilities [23], and a twin study further indicated shared genetic influences on RD with verbal short-term memory (STM) and WM [24].

To our knowledge, only one study to date has examined the aetiological sharing between ADHD symptoms, RD and specific cognitive processes. Willcutt et al. studied a population sample of 457 twin pairs, aged 8–18, from the Colorado Learning Disabilities Research Centre study [4]. Genetic factors underlying slow processing speed (measured as MRT in symbol search and picture identification tasks) captured a substantial proportion of shared genetic risks between ADHD symptoms and RD, whereas a significant proportion of genetic influences on inhibition or WM were independent of the genetic covariance between reading and inattention symptoms [4]. These findings require replication and extension into further cognitive measures.

Using multivariate model-fitting analyses on a large population twin sample, with a tightly defined age range (7–10 years), this study aims to investigate which cognitive impairments previously linked to either ADHD or RD or both (specifically RTV, response inhibition, verbal STM and WM, and choice impulsivity [3]), independent of IQ effects, underlie the co-occurring symptoms. Specifically, we address three key questions: i) Which cognitive impairments are associated with both ADHD symptoms and RD? ii) To what extent do these cognitive measures (the identified cognitive variables, RD, and ADHD symptoms) share genetic influences? iii) To what extent does a shared cognitive impairment capture the shared genetic risk between ADHD symptoms and RD?

## Materials and Methods

### Ethics statement

The study was conducted in accordance with the Declaration of Helsinki and was approved by the Institute of Psychiatry Research Ethics Committee. Parents of all participants gave written consent to the study procedure before their participation.

### Sample and Procedure

Participants are members of the Study of Activity and Impulsivity Levels in children (SAIL), a general population sample of twins aged between 7 and 10 years. They were recruited from the Twins' Early Development Study (TEDS; [25], a birth cohort study in which parents of all twins born in England and Wales during 1994–1996 were invited to enroll. TEDS families were invited to take part if they fulfilled SAIL project criteria, including White European ethnic origin (to reduce population heterogeneity for molecular genetic studies); no extreme pregnancy or perinatal difficulties, specific medical syndromes, chromosomal anomalies or epilepsy; and not on stimulant or other neuropsychiatric medications (see [26] for a full list of inclusion criteria).

Of the 1,230 suitable families contacted, 672 families (55%) agreed to participate. Thirty-two children were subsequently excluded due to: IQ <70, epilepsy, autism, obsessive-compulsive or other neurodevelopmental disorder, illness during testing or placement on stimulant medication for ADHD. The final sample consisted of 1312 individuals: 257 monozygotic (MZ) twin pairs, 181 same-sex dizygotic (DZ) and 206 opposite-sex DZ twin pairs, as well as 24 singletons coming from pairs with one of the twins excluded. Data for the 24 singleton twins were also used in the structural equation modeling [27].

The families visited the research centre for the assessments. Two testers assessed the twins simultaneously in separate testing rooms. The tasks were administered in a fixed order as part of a more extensive test session, which in total (including breaks) lasted approximately 2.5 hours. The mean age of the sample was 8.83 (SD  = 0.67), and half of the sample were female (N = 663, 50.5%). Children's IQs ranged from 70 to 158 (M = 109.34, SD  = 14.72).

### Measures

#### ADHD Ratings

Parent and teachers were asked to complete the Long Versions of Conners' Parent Rating Scale [28] and the Long Version of Conners' Teacher Rating Scales [29]. To obtain a composite score for ADHD symptoms, ADHD inattention and hyperactivity-impulsivity symptoms were obtained using the summed parent and teacher ratings on the 9-item inattentive DISM-IV subscales and the 9-item hyperactivity-impulsivity DSM-IV subscales, respectively, consistent with our previous studies that examined cognitive measures in relation to ADHD traits in this sample [3], [30]–[32]. Teacher ratings were missing for 151 individuals and parent ratings for two individuals.

#### Reading difficulties

Reading Difficulties Questionnaire (RDQ) is a subscale of the Colorado Learning Difficulties Questionnaire [33]. This six-item parent rating scale is part of an instrument screening for learning disorders. On a scale that ranges from 1 (never/not at all) to 5 (always/a great deal), parents reported the extent of their child's difficulties with spelling, learning letter names, sounding words out, and to what extent their child reads slowly, below expectancy level or has required extra help at school. The total score ranges from 5 to 30, with higher scores indicating greater difficulties with reading. This scale has been shown to have excellent internal consistency (mean Cronbach's α = 0.90) and high inter-rater (*r* = 0.83) and one-year test-retest (*r* = 0.81) reliabilities [34]. RDQ has shown strong correlations with other objective reading and spelling measures (average *r* = 0.64), but low correlations with measures of other learning difficulties (*r* = 0.07 to 0.02), which attest to its good criterion and discriminant validity [34]. Moreover, RDQ scores have demonstrated moderate to high heritability (*h* ^2^ = 53 to 83%) and high genetic correlations (0.71 to 0.89) with a composite measure of reading performance [5], [35].

#### Wechsler Intelligence Scales for Children, Third Edition (WISC-III; [36])

The vocabulary, similarities, picture completion and block design subtests from the WISC-III were used to obtain an estimate of the child's IQ (prorated following procedures described by [37]). The digit span subtest from the WISC-III was administered to obtain digit span forward (DSF) and digit span backward (DSB) [36], which measure verbal STM and WM, respectively.

#### The Go/No-Go task [38], [39]

On each trial, one of two possible stimuli appeared for 300 ms in the middle of the computer screen. The participant was instructed to respond only to the ‘go’ stimuli and to react as quickly as possible, but to maintain a high level of accuracy. The proportion of ‘go’ stimuli to ‘no-go’ stimuli was 4∶1. The participants performed the task under three conditions (slow, fast and incentive), matched for length of time on task. Herein we present data from the slow condition, which had an inter-stimulus interval (ISI) of 8 s and consisting of 72 trials, and the fast condition, with an inter-stimulus interval (ISI) of 1 second and consisting of 462 trials. The order of presentation of the slow and fast conditions varied randomly across participants. We focus here on two variables obtained from the task: CE and RTV.

#### The Fast Task [40], [41]

The baseline condition, with a foreperiod of 8 s and consisting of 72 trials, followed a standard warned four-choice RT task. A warning signal (four empty circles, arranged side by side) first appeared on the screen. At the end of the foreperiod (presentation interval for the warning signal), the circle designated as the target signal for that trial was filled (coloured) in. The participant was asked to make a compatible choice by pressing the response key that directly corresponded in position to the location of the target stimulus. Following a response, the stimuli disappeared from the screen and a fixed inter-trial interval of 2.5 s followed. Speed and accuracy were emphasised equally. If the child did not respond within 10 s, the trial terminated. A comparison condition with a fast event rate (1 second) and incentives followed the baseline condition [41]. Herein we focus on RTV, obtained from the baseline condition.

To limit the total number of variables and to create psychometrically robust variables that would enable direct comparisons to our previous findings using the same tasks in a clinically diagnosed sample [8], the summed unstandardized scores of RTV were obtained across the baseline conditions of the Go/No-Go and Fast tasks [30], [40]. A composite measure of CE was obtained by summing the raw CE scores from both the baseline (slow) and the fast conditions of the Go/No-Go task [40].

#### The Maudsley Index of Childhood Delay Aversion [26], [31]

Two conditions, each with 20 trials, were administered. In each trial, the child had a choice between a smaller-immediate reward (one point involving a 2-second pre-reward delay) and a larger-delayed reward (two points involving a 30-second pre-reward delay). In the no post-reward delay condition, choosing the small reward led immediately to the next trial, reducing the overall length of the condition. In the post-reward delay condition, choosing the small reward led to a delay period of 30 seconds, and choosing the large reward led to a delay period of 2 seconds before the next trial; therefore, the overall delay was constant and independent of choice made. The order of the two conditions was randomly chosen for each twin. Choice impulsivity (CI) was calculated as the number of times the smaller-immediate reward was selected in the no post-reward delay condition, controlling for total number of trials attempted.

### Statistical analyses

#### Structural equation models

The structural equation modeling program Mx was used [27]. Models were fitted to IQ-, age- and sex-regressed unstandardized residual summed scores, which were transformed to minimize skewness using the optimized minimal skew command in Stata (version 10.0; StataCorp, 2007). All estimates are provided with 95% confidence intervals (the inclusion of zero indicates non- significance). The relative goodness of fit of the competing hierarchical (or nested) models was assessed using a likelihood ratio test. Mx handles missing data by using raw maximum likelihood estimation to calculate a likelihood statistic for each missing observation based on the observed variance/covariance matrix. Therefore, participants with missing data were included in the analysis.

#### Univariate genetic models

Univariate genetic analyses use twin-pair correlations for a trait and, on the basis that MZ twins share 100% of their segregating alleles and DZ twins share 50% of additive genetic influences as well as 25% non-additive genetic influences, partition the phenotypic variance of the measures into additive genetic (A), dominance (D) or shared environmental (C), and non-shared environmental (E) effects. Any possible measurement error is subsumed under the E effects [42]. Greater phenotypic similarity between MZ twins, compared to DZ twins, suggests genetic influences on trait variance. If the phenotypic similarity of MZ twins is more than twice that of DZ twins, this suggests the presence of D, otherwise only A is suggested. DZ twin correlations greater than half the MZ twin correlations suggest the presence of C. The extent to which MZ twins are not 100% concordant for a trait reflects E [42]. As multivariate models have increased power over univariate models [43], we do not present parameter estimates from univariate models. Univariate modeling was used to inform the choice of parameters for the multivariate models (e.g. the choice of C or D parameters) and to test for sex effects.

#### Sex effects

Scalar differences for reading difficulties and inattention were observed. Scalar sex differences are found where only unstandardized A, C/D and E estimates differ (but standardized estimates are the same), due to variance differences in the trait distribution between males and females. Therefore, in the multivariate modeling, male phenotypic variances for these traits were pre- and post- multiplied by a scaling factor. As there are no significant qualitative or quantitative differences in variance components between the sexes, MZ and DZ correlations are not presented for each sex. However, given the scalar differences between the sexes, means and standard deviations are broken down into sex- and zygosity- specific groups (Table S1).

#### Parameter selection for the multivariate models

In the univariate analyses, an AE model provided the best fit for DSF, DSB and RTV, while ADE models (with scalar sex differences) fitted best for inattention and RD (as we would predict from the MZ: DZ ratios of cross-twin correlations for these traits; Table S1). In this study, as we lacked sufficient power to distinguish between A and D effects, we model broad-sense genetic (G) influences that combines both A and D effects. As there were no qualitative or quantitative sex differences in the univariate analyses beyond scalar differences, and due to the computational intensity of modeling sex effects and additional power issues [27], only scalar differences between males and females were allowed in the multivariate models.

#### Multivariate genetic models

**Figure 1:**
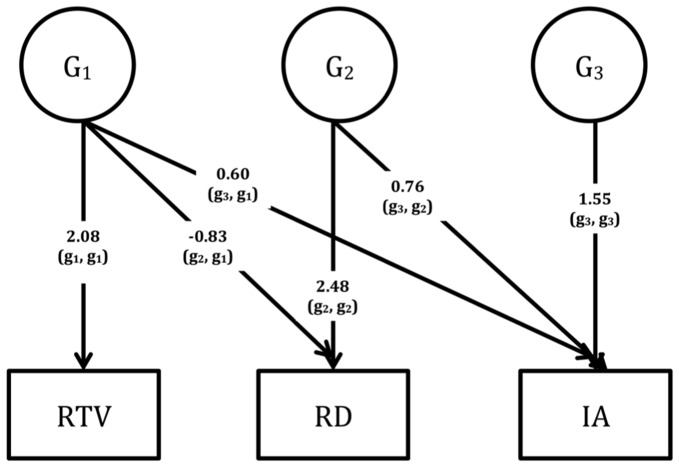
Unstandardised parameter estimates (G1–G3) from the Cholesky decomposition across reaction time variability (RTV), reading difficulties (RD) and inattention (IA).

**Figure 2:**
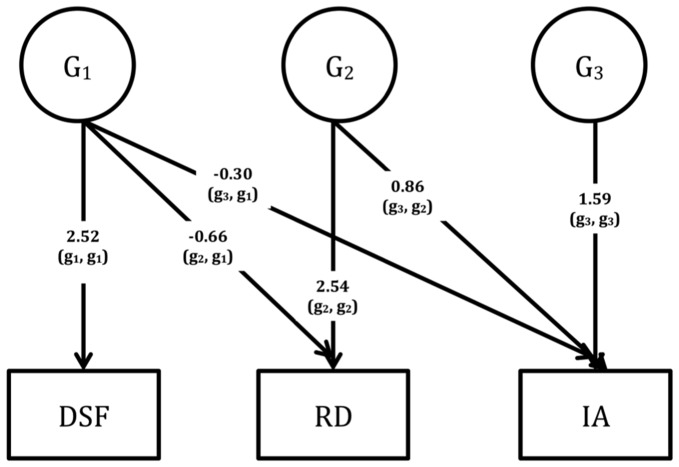
Unstandardised parameter estimates (G1–G3) from the Cholesky decomposition across digit span forward (DSF), reading difficulties (RD) and inattention (IA).

**Figure 3:**
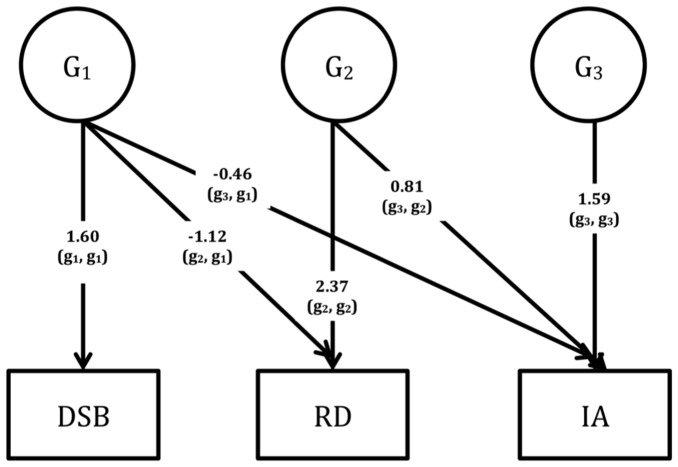
Unstandardised parameter estimates (G1–G3) from the Cholesky decomposition across digit span backward (DSB), reading difficulties (RD) and inattention (IA).

Multivariate genetic analyses use the power given by the MZ: DZ ratio of cross-twin cross-trait correlations to decompose the covariation between traits into G and E influences [42]. A Cholesky decomposition was fitted to the data; and to estimate the extent to which the genetic (G) and environmental (E) factors overlap (i.e. the genetic (r_g_) and environmental (r_e_) correlations), the standardized (correlated factors) solution of the Cholesky was used to avoid giving precedence to any one latent variable where the ordering of measured variables was arbitrary [44]. The total covariance model is than given by G*r_g_*G' + E*r_e_*E'. In the Cholesky, a triangular decomposition is used to decompose the variance in each phenotype and covariance between the phenotypes into broad sense genetic (G1-G3; Figures 1–3) and unique environmental (E1–E3) influences. Since Cholesky decompositions require an a priori justification of variable order where they contain more than three variables (based on, for example, temporality within longitudinal data), and our data were cross-sectional, we ran three separate Cholesky models to determine the extent to which the covariation between ADHD symptoms and RD is independent of cognitive measures of RTV, verbal STM and WM. RTV/STM/WM (i.e. the objectively measured cognitive process) was entered as the first variable in the Cholesky model, with the rating scale data (RD and inattention) as second and third variables.

We regressed IQ from all cognitive variables and reading difficulties to ensure that we controlled for any mediating effects of IQ that were not the focus of the present analyses, consistent with our previously adopted approach [8]. Our previous analyses on the current [30] and a separate [8] sample have indicated that the majority of genetic influences shared between ADHD and cognitive variables are independent of those shared with IQ [30], [45].

## Results

### Which cognitive impairments are associated with both ADHD symptoms and RD?

The maximum-likelihood correlations adjusting for familial structure indicated that a correlation between inattention and reading difficulties (RD) of 0.48 (Table 1). Intra-class partial correlations from STATA (which used the ‘cluster command to control for the family structure) indicated that this dropped to r = 0.39, r = 0.42 and r = 0.41 when controlling for RTV, digit span forward (DSF) and digit span backward (DSB), respectively. RTV, DSF and DSB were significantly associated with both ADHD inattention symptoms and RD (Table 1). CE and choice impulsivity (CI) were not associated with RD, and only RTV and CE showed significant correlations with hyperactivity-impulsivity. Therefore, we only included RTV, DSF, DSB, inattention and RD in further genetic analyses.

**Table 1:** Phenotypic correlations across inattention (IA), hyperactivity-impulsivity (H-I), reading difficulties (RD), reaction time variability (RTV), commission errors (CE), choice impulsivity (CI), digit span forward (DSF) and digit span backward (DSB).

	IA	H-I	RD	RTV	CE	CI	DSF
**H-I**	.59*						
**RD**	.48*	.17*					
**RTV**	.26*	.16*	.18*				
**CE**	.13*	.09*	.06	.12*			
**CI**	.14*	.08	.04	.08*	.06		
**DSF**	−.11*	−.06	−.15*	−.06	.01	−.06	
**DSB**	−.15*	−.05	−.18*	−.14*	−.05	−.09*	.29*

*p<0.01.

### To what extent do inattention, RD, RTV, verbal STM and WM share genetic /unique environmental influences?

Genetic factors accounted for around 60%, 30% and 40% of the variances on DSF, DSB and RTV, respectively (Table 2). All cognitive variables showed significant genetic correlations (r_g_) with inattention symptoms and RD. The unique environmental correlations (which also includes measuring error) (r_e_) were not significant between any cognitive variables and RD (all r_e_s <0.05). Inattention showed significant r_e_ only with RTV but not with DSF or DSB. There was substantial genetic overlap between DSB and DSF (r_g_ = 0.63), but the genetic overlap between DSB and RTV was not significant (indicated by confidence intervals overlapping zero).

**Table 2:** Standardised parameter estimates (with 95% confidence intervals) from the correlated factor model across digit span forward (DSF), digit span backward (DSB), reaction time variability (RTV), reading difficulties (RD) and inattention (IA).

	DSF	DSB	RTV	RD	IA
**Genetic influences**					
**DSF**	**0.59 (0.51, 0.66)**	*0.25 (86%)*	*	−*0.15 (97%)*	−*0.09 (91%)*
**DSB**	0.63 (0.46, 0.80)	**0.27 (0.17, 0.36)**	−*0.06 (43%)*	−*0.19 (97%)*	−*0.09 (56%)*
**RTV**	*	−0.17 (−0.37, 0.05)	**0.42 (0.33, 0.51)**	*0.16 (89%)*	*0.15 (63%)*
**RD**	−0.24 (−0.36, −0.12)	−0.46 (−0.50, −0.28)	0.32 (0.18, 0.46)	**0.63 (0.55, 0.69)**	*0.67 (0.50, 0.82)*
**IA**	−0.17 (−0.31, −0.03)	−0.24 (−0.44, −0.03)	0.32 (0.18, 0.46)	0.52 (0.46, 0.60)	**0.52 (0.40, 0.62)**
**Unique environmental influences**					
**DSF**	**0.41 (0.34, 0.49)**	*0.04 (14%)*	*	−0.004 (3%)	−0.009 (9%)
**DSB**	0.08 (−0.02, 0.19)	**0.73 (0.64, 0.83)**	−*0.08 (57%)*	−*0.005 (3%)*	−*0.07 (44%)*
**RTV**	0.13 (0.02, 0.24)	−0.12 (−0.22, −0.02)	**0.58 (0.49, 0.67)**	*0.02 (11%)*	*0.09 (37%)*
**RD**	−0.01 (−0.13, 0.12)	−0.01 (−0.12, 0.10)	0.04 (−0.07, 0.15)	**0.37 (0.31, 0.45)**	***0.33 (0.18, 0.50)***
**IA**	−0.02 (−0.13, 0.12)	−0.11 (−0.22, 0.01)	0.18 (0.06, 0.29)	0.34 (0.21, 0.46)	**0.48 (0.38, 0.59)**

The heritability (g^2^) and unique environmental variances (e^2^) are indicated as bold along the diagonal. The genetic and unique environmental correlations (and 95% confidence intervals) between pairs of variables are given below the diagonal. The contributions of genetic and unique environmental influences to the phenotypic correlations between variables are given above the diagonal (and the percentage of the phenotypic correlations due to each aetiological factor).

*Not interpreted due to a lack of phenotypic associatio.

### To what extent does a shared cognitive impairment capture the shared genetic risk between ADHD symptoms and RD?

Using the Cholesky decomposition, we calculated the sum of genetic (G) influences underlying the G covariance between inattention and RD that were not shared with RTV (G_2,2_ x G_3,2_ in Figure 1) as a percentage of the total genetic covariance between inattention and RD (G_2,1_ x G_3,1_ + G_2,2_ x G_3,2_). This led us to deduce that 72% of the genetic overlap between inattention and RD was driven by shared genetic influences that are independent of those underlying RTV. Using the same method, we found that 89% and 79% of the genetic covariance between inattention and RD was independent of the genetic influences underlying DSF and DSB, respectively (Figure 2 and Figure 3). Since there was no significant overlap in unique environmental influences between RD and any of the cognitive variables, we did not interpret the E findings from the Cholesky decomposition.

## Discussion

In genetic model fitting analyses on a population sample of twins aged 7–10, we identified three cognitive processes – reaction time variability (RTV), verbal working memory (WM) and verbal short-term memory (STM) – that showed significant phenotypic and genetic associations with both inattention symptoms and reading difficulties (RD). As the genetic influences on RTV separated from those on the memory measures, we further examined, for each cognitive variable in turn, the extent to which it captured the shared genetic risk between inattention and RD. While STM captured only 11% of the shared genetic risk between inattention and RD, the estimates increased somewhat for WM (21%) and RTV (28%); yet most of the genetic sharing between inattention and RD remained unaccounted for in each case.

Response inhibition (CE) and choice impulsivity (stronger preference for smaller-immediate rewards) were not significantly associated with RD, and therefore did not emerge as important cognitive processes that underlie the co-occurrence between ADHD symptoms and RD. Of the two ADHD symptom domains, the associations were largely limited to inattention, with only RTV and CE showing significant, though low, correlations with hyperactivity-impulsivity; the association between hyperactivity-impulsivity and RD was also low (0.17), though significant. Overall, the pattern of results further supports the partial etiological separation of the two ADHD symptom domains [46], [47].

We observed no phenotypic association between STM and RTV. Despite some evidence of a phenotypic association between WM and RTV (r_ph_ = 0.14), there was no significant genetic overlap between them. These findings of the genetic risk factors underlying verbal memory processes separating from the genes that increase the susceptibility for increased RTV are consistent with the aetiological separation between top-down executive functioning in working memory (WM) and measures of intra-individual variability previously reported in a clinical ADHD and control sibling-pair sample [9].

The strengths of studying twin pairs from the general population lie in the ability to examine the two ADHD symptom domains separately and free from potential referral effects [48]. A limitation of the present study is that, despite a large sample of over 1300 twins, we lacked sufficient power to distinguish between additive (A) and dominance (D) genetic effects in the present multivariate analyses on this set of variables where univariate analyses suggested D effects for only two of them [49]. We therefore modelled ‘broad sense heritability’ (A+D influences) only. Future replication of these findings in larger samples is therefore important. Another limitation of this study is the inclusion of only parent ratings of RD; future studies should replicate these findings using examiner-administered measures of reading.

In this study we did not have a measure of processing speed, which has previously been identified as important for the association between RD and ADHD [4]. While processing speed measured in previous studies [2], [4] may capture some elements of mean reaction time (MRT), the aetiology of these two measures cognitive processes are likely to be different. Compared to MRT and RTV obtained from simple RT tasks, where the underlying genetic influences shared with ADHD largely separate from those ADHD shares with IQ [30], [45], processing speed measures are derived from IQ measures and capture higher cognitive processes such as matching and ordering objects; thus, our findings are not directly comparable with those in the previous study [4]; the associations between processing speed, MRT and RTV remain for future studies to explore further. As executive functions continue to develop throughout childhood and adolescence, their overlap with ADHD and RD should also be examined in future research from a developmental perspective across a wide age range.

In this study, we examined the aetiological relationship between ADHD and reading difficulties beyond general cognitive ability (IQ) to specific cognitive impairments associated with ADHD. We identified three cognitive processes (RTV, STM and WM) that share significant genetic influences with both inattention and reading difficulties. These findings inform and guide future research on the partly shared pathways from genetic influences to cognitive processes that underlie the observed association between ADHD and RD. Future studies should extend the investigation into additional cognitive measures, as well as further objective measures of reading. Longitudinal studies will be essential to investigate the developmental pathways and direction of causality.

## Supporting Information

Table S1 **Twin correlations, means and standard deviations across inattention (IA), reading difficulties (RD), reaction time variability (RTV), digit span forward (DSF) and digit span backward (DSB).**
(DOCX)
