# Correction: Shared Cognitive Impairments and Aetiology in ADHD Symptoms and Reading Difficulties

**DOI:** 10.1371/journal.pone.0115924

**Published:** 2014-12-12

**Authors:** The PLOS ONE Staff

The second author’s last name is misspelled. The correct name is: Alexis C. Frazier-Wood. The correct citation is given below.

Cheung CHM, Frazier-Wood AC, Asherson P, Rijsdijk F, Kuntsi J (2014) Shared Cognitive Impairments and Aetiology in ADHD Symptoms and Reading Difficulties. PLoS ONE 9(6): e98590. doi:10.1371/journal.pone.0098590
