# Supplementary material for: Shared Cognitive Impairments and Aetiology in ADHD Symptoms and Reading Difficulties
Source: PLoS One. 2014 Jun 2;9(6):e98590. doi: 10.1371/journal.pone.0098590 (PMC4041781; doi:10.1371/journal.pone.0098590)
Supplement: Table S1 — Twin correlations, means and standard deviations across inattention (IA), reading difficulties (RD), reaction time variability (RTV), digit span forward (DSF) and digit span backward (DSB). (DOCX) [file pone.0098590.s001.docx]

**Table S1. Twin correlations^a^ (with 95% confidence intervals), means and standard deviations^b^  across inattention (IA), reading difficulties (RD), reaction time variability (RTV), digit span forward (DSF) and digit span backward (DSB).**

| **Cross-twin correlations** | | |  |  |  |  |  |  |  |  |
| --- | --- | --- | --- | --- | --- | --- | --- | --- | --- | --- |
|  | **IA** |  | **RD** |  | **RTV** |  | **DSF** |  | **DSB** |  |
|  | **MZ** | **DZ** | **MZ** | **DZ** | **MZ** | **DZ** | **MZ** | **DZ** | **MZ** | **DZ** |
| **IA** | **.56 (.47, .68)** | .08 (-.02, .19) |  |  |  |  |  |  |  |  |
| **RD** | **.36 (.24, .47)** | .07 (-.03, .17) | **.67 (.60, .74)** | **.16 (.06, .29)** |  |  |  |  |  |  |
| **RTV** | **.23 (.10, .34)** | -.03 (-.13, .08 ) | **.18 (.06, .30)** | .06 (-.04, .17) | **.44 (.33, .54)** | **.21 (.11, .31)** |  |  |  |  |
| **DSF** | **-.15 (-.28, -.03)** | -.02 (-.09, .12) | **-.15 (-.27, -.03)** | -.07 (-.17, .04) | -.11 (-.23, .02) | .01 (-.10, .11) | **.59 (.50, .66)** | **.28 (.18, .37)** |  |  |
| **DSB** | -.02 (-.15, .11) | .08 (-.02, .18) | **-.21 (-.33, -.10)** | -.02 (-.12, .08) | -.10 (-.22, .02) | .04 (-.07, .14) | **.37 (.26, .47)** | .10 (.00, .20) | **.29 (.18, .40)** | **.11 (.01, .21)** |
| **Male** mean (SD) | 12.70 (8.95) | 7.79 (6.51) | 10.79 (6.33) | 11.47 (6.66) | 619.06 (350.81) | 631.01 (376.52) | 7.74 (1.80) | 7.43 (1.58) | 4.36 (1.42) | 4.23 (1.30) |
| **Female** means  (SD) | 14.25 (11.14) | 9.06 (7.88) | 9.90 (4.75) | 9.82 (5.06) | 629.94 (354.15) | 628.04 (359.12) | 7.74 (1.60) | 8.05 (1.72) | 4.52 (1.46) | 4.62 (1.40) |

Significant correlations in **bold**

^a^ estimated using maximum likelihood estimation

^b^ Raw score
